# Cod Liver Oil Substitute

**Published:** 1918-11

**Authors:** 


**Cod Liver Oil Substitute.** Col. J. S. Purdy, Brit. Med. Jour., calls attention to the mutton bird or smoky petrel whose migration is between the Antarctic and New Zealand and Australia. The oil is used by the natives and the author has employed it in tuberculosis. (Note: We have long been sceptic, first of the special value of cod liver oil, secondly, of the value of a high fat or any other unbalanced ration in tuberculosis, although a moderate excess of nourishment is demanded. However, when fat has shown its importance both as a food and as a war material, by the shortage in Germany any source of fat is welcome. E.)

